# Mental Fatigue Is Associated with Subjective Cognitive Decline among Older Adults

**DOI:** 10.3390/brainsci13030376

**Published:** 2023-02-21

**Authors:** Qianqian Zhang, McKenna Angela Sun, Qiuzi Sun, Hua Mei, Hengyi Rao, Jianghong Liu

**Affiliations:** 1 School of Nursing and Medicine, University of Pennsylvania, Philadelphia, PA 19104, USA; 2 Longhua Hospital, Shanghai University of Traditional Chinese Medicine, Shanghai 201203, China; 3 Shanghai University of Medicine and Health Sciences, Shanghai 201318, China

**Keywords:** aging, mental fatigue, subjective cognitive decline, sex difference, old adult

## Abstract

Both Subjective Cognitive Decline (SCD) and mental fatigue are becoming increasingly prevalent as global demographics shifts indicate our aging populations. SCD is a reversible precursor for Alzheimer’s disease, and early identification is important for effective intervention strategies. We aim to investigate the association between mental fatigue—as well as other factors—and SCD. A total of 707 old adults (aged from 60 to 99) from Shanghai, China, participated in this study and completed self-reported instruments covering their cognitive and mental status as well as demographic information. Mental fatigue status was assessed by using four items derived from the functional impairment syndrome of the Old Adult Self Report (OASR). SCD was assessed by using the Memory/Cognition syndrome of OASR. A total of 681 old adults were included in the current study. The means of SCD significantly differed between each group of factors (age, gender, and mental fatigue). The general linear regression models showed that SCD increased with age, females scored higher than males, and SCD was positively associated with mental fatigue factors including difficulty getting things done, poor task performance, sleeping more, and a lack of energy among old adults. The study also found that SCD is negatively associated with the high-income group among young-old (aged from 60 to 75) males and associated with good marital/living status with the companion of spouses/partners among young-old females. These results suggest that gender, income level, marital/living status, and mental fatigue are crucial factors in preventing SCD among old adults and are pivotal in developing early intervention strategies to preserve the mental health of an increasingly aging population.

## 1. Introduction

In the past several decades, the global population demographic has shifted to becoming increasingly elderly [1]. By 2050, it is estimated that one-sixth of the global population will be over the age of 65, totaling approximately 1.5 billion people [2]. Specifically, China faces a rapidly aging population compared to other countries [3]. By 2040, 402 million people (28% of the total Chinese population) are predicted to be over the age of 60 [4]. A recent study estimated a 15.5% prevalence of mild cognitive impairment among Chinese older adults, representing 38.77 million people in China; additionally, 15.07 million people aged 60 years and older in China have dementia [5]. This poses a dire issue for China, as aging is closely associated with cognitive decline [6]. Cognitive decline can range from mild cognitive impairment to dementia. Cognitive impairment is when a person has problems with cognitive abilities, such as memory, awareness, and judgment [7]. Mild cognitive impairment (MCI) can cause cognitive changes that are serious enough to be noticed by the afflicted individual and by family members and friends, but may not affect the individual’s ability to carry out daily activities, whereas severe levels of impairment can lead to the inability to live independently [7]. Subjective Cognitive Decline (SCD) is a form of cognitive impairment referring to the self-reported experience of worsening or more frequent confusion or memory loss [8]. It has been recently suggested to be a preclinical stage to the onset of Alzheimer’s disease and is associated with other mental disorders, such as depression and anxiety [9,10,11,12,13]. Therefore, SCD could be an ideal target for early therapeutic intervention [13].

The prevalence of SCD is associated with female gender—a national cross-section study reported 23.91% old adults in the female gender group have SCD, compared to 15.86% of the male gender group in relation to Chinese old adults [5]. The incidence of SCD is correlated with an increase in age—the Behavioral Risk Factor Surveillance System (BRFSS) reported that 11.7% of U.S. adults above the age of 64 experience SCD compared to 10.8% of U.S. adults between ages 45–64 [8]. Yan et al. reported higher prevalence of cognitive impairment of older Chinese old adults (aged 80 to 105) than younger ones (aged 65–79) [14]. However, less is known about their progression solely within different age groups in old adults. Studies classify elderly age groups in different manners. Forman et al. used 70-years-old as the classification threshold, while Ouchi et al., Lee et al., and Alterovitz and Mendelsohn defined people from 65–75 as pre-old (younger-old) age and those above 75 as old (old-old) age. [15,16,17,18]. The Cohort Studies of Memory in an International Consortium (COSMIC) estimated SCD prevalence across elderly age group subsets and found that those aged from 65–74 years had a generally lower incidence of SCD compared to those aged from 60–64 years and 75+ years [19]. Cognitive deficits can additionally lead to other health problems. Yuan et al. found that old adults with cognitive impairment aged over 75 years have a higher risk of death and of suffering from frailty in one year compared with those aged between 65–74 years [20]. Therefore, current studies define old adults aged between 60–75 as young-old and those aged above 75 as old-old adults, and it is important to investigate the association between SCD within each age category.

In addition to age and gender, the prevalence of SCD has also been shown to be correlated with differences in household factors, education level, ethnicity, mental distress, and functional difficulties [8]. Currently, there is no universally accepted standard for the measurement of SCD. A holistic review of SCD measurement tools indicated inconsistent conclusions about SCD as a result of scale variability among studies, and a consensus on the most suitable scale has yet to be reached [21,22]. Therefore, additional research is necessary to both explore the related risk factors for SCD and develop comprehensive screening tools for SCD to improve the health and well-being of older adults.

Mental fatigue is a psychobiological state with subjective, behavioral, and physiological consequences for an individual, such as heightened feelings of tiredness, increased reaction times, and reduced physical energy [23]. Mental fatigability, defined as an inability to sustain engagement in mentally taxing activities, is a frequent problem among elderly individuals but is often unaddressed by the medical community [24]. Perceived mental fatigability has been shown to significantly increase with age and to be significantly higher in women than men [25]. In addition, mental fatigue carries consequences for other areas of mental health (e.g., action regulation, motivation) and impairs physical activity levels [26,27]. The measurement of mental fatigue is not standardized, as studies commonly utilize various self-report techniques to gauge mental fatigability, yet there has also been a shift to reaction time-based tests [23,24]. In this study, the Older Adult Self-Report (OASR), which measures diverse aspects of adaptive functioning based on self-perception, was utilized to measure mental fatigue and other potential SCD risk factors because of its validity among Chinese-speaking older populations [28,29].

In recent years, there has been increasing interest in the association between various mental stresses and SCD. Individuals with stress-related exhaustion disorders tend to have significantly more subjective cognitive complaints, an indicator of SCD, compared to their healthy counterparts [30]. Acceptance therapy for hematological cancer survivors is strongly associated with reduced mental fatigue and reduced SCD [31]. Additionally, during the COVID-19 pandemic, confinement stress served the greatest effect on predictors (e.g., negative emotions, generalized anxiety) for increased SCD in Slovenian adults [32]. The Long Life Family Study showed that severe mental fatigue among the elderly (ages 60+) was significantly correlated with poorer cognition and increased depressive symptomology [25]. However, a connection between mental fatigue and SCD in the elderly has yet to be explored. This gap in the literature is crucial because its closure may provide timely interventions and treatments for the well-being of an increasingly large elderly population.

Cognitive deficits present in SCD are more easily reversible compared to later stages, such as mild cognitive impairment and Alzheimer’s disease, making SCD a crucial stage for early intervention [33]. Given the current literature gap concerning a direct association between mental fatigue and SCD in elderly individuals and given the necessity to identify risk factors for SCD, this cross-sectional study aims to investigate whether differences in SCD exist between different comparison groups such as age and gender and whether mental fatigue problems are risk factors for SCD among older adults in Shanghai. We hypothesized that (1) SCD prevalence would be higher in females and higher with age and that (2) mental fatigue problems are significant indicators of SCD for old adults and are significant when stratified via age and gender groups. This study has important implications for developing early treatment strategies to improve the cognitive outlook and psychophysical well-being of an increasingly aging population.

## 2. Methods

### 2.1. Participants and Procedure

The community sample was composed of 755 old adults, aged above 59 years old, without significant mental or physical disabilities, recruited from 25 local communities in the metropolitan city of Shanghai using convenience sampling and community sampling strategies [34]. The nursing research team from Shanghai Longhua Hospital and Shuguang Hospital conducted the recruitment process in November 2017. The recruitment advertisements were posted two weeks in advance of the healthcare educational event to inform individuals about the purpose of the study. Compensations of 100 Chinese Yuan were given as incentives to individuals who participated in the research. The surveys were administered by 150 interviewers, including 40 nursing supervisors from Shanghai Longhua Hospital and 110 interns from Shanghai Shuguang Hospital. Nurses, who were given the questionnaire by nursing supervisors, distributed the questionnaires to the older adults, including residents in their neighborhood. Training workshops were held to standardize the interview procedure. The respondent was handed a copy of the survey to view while the interviewers read each item aloud and wrote the respondent’s answers on a second copy. If it was clear that the respondent could complete the items independently, the respondent was given the option of writing his/her own answers. Informed consent was obtained from all participants and institutional review board approval was obtained from the ethics committees for research at both Shanghai Shuguang Hospital and Shanghai Longhua Hospital (ethics approval number: 2020-GZR-02-053X). Through collaborations between Shuguang Hospital, Longhua Hospital, and local community centers, 707 participants (94% response rate) completed cross-sectional surveys.

### 2.2. Measure

Old adults’ mental fatigue and SCD were assessed using the syndrome scales of the Old Adult Self Report (OASR). The OASR is a standardized form developed by Dr. Thomas M. Achenbach and Dr. Paul A. Newhouse via which older adults report their adaptive functioning, personal strengths, behavioral, emotional, and social problems, and their use of cigarettes, alcohol, and drugs [28]. An extensive survey was developed to gather additional demographic information. The syndromes of OASR have been demonstrated to fit the data across 20 different societies, such as China, Brazil, and Germany [35]. The Chinese version of OASR has been demonstrated to be applicable to adults to access their mental health in clinical and research settings among Chinese-speaking older adult populations [34,35].

Mental fatigue symptoms were measured using four items selected from the functional impairment syndrome scale under the OASR. These four items are Difficulty getting things done, Poor task performance (Poor task), Sleeps more, and Lacks energy. All items were rated on a three-point scale (0 = not true, 1 = sometimes true, and 2 = often true) based on the preceding 2 months.

SCD was assessed using the Memory/Cognition Problems Syndrome Scale in the OASR, following the scoring methodology mentioned above. These 9 items include: I have trouble concentrating or paying attention (Cannot concentrate), I feel confused or in a fog (Confused), I forget people’s names (Forgets names), I have trouble finishing things I should do (Cannot finish), I have trouble making decisions (Trouble w. decisions), I have trouble talking (Cannot talk), I have trouble remembering things I am told (Cannot remember), If I do not write things down, I forget them (Forgets if not written down), and I worry too much about my memory (Worries abt. memory). SCD scores were obtained by summing the items under each syndrome, presented in a normalized T-score form (M = 50, SD = 10). Higher scores indicated a higher level of SCD problems.

Demographic factors include age, gender (male/female), education level (high-school equivalence and above is defined as the high-education group, high-school degree unearned is defined as the low-education group), income level (total monthly income of RMB 3000 or higher is defined as high-income, and below RMB 3000 is defined as low income), and marital/living status (married and living with spouses, unmarried but living with partners are defined as one group; unmarried, divorced, widowed, and not living with spouse/partner in the past two months were defined as the other group). The samples were divided by age into 2 groups for analysis: young-old (aged from 60 to 74) and old-old (aged 75 and above).

### 2.3. Data Analyses

The data analysis was performed with RStudio. While measuring mental fatigue and SCD, the 26 individuals who had more than 1 missing item for syndromes were excluded from the analysis procedure; otherwise, the missing item was filled using 0 (N = 6 of 681), leaving 681 valid data records. The sample was broken down into age and gender cohorts. Frequencies, percentages, means, and standard deviations were used to describe the sample characteristics and each cohort. The mean differences between age/gender/mental fatigue symptoms and SCD were measured via one-way ANOVA, controlling for potential confounding demographic effects. To interpret the independent effect of mental fatigue symptoms on cognitive decline, the general linear regression models were performed for all community samples and separately by age and gender groups, controlling for all demographic variables (gender, age, education level, income level, and marital/living status). A *p*-value of less than 0.05 was considered statistically significant.

## 3. Results

### 3.1. Sample Characteristics

Among 681 old adults, about three-fourths of participants (N = 508) were young-old adults, one-fourth were old-old adults (N = 173), 45% (N = 306) were males, and 55% (N = 375) were females. The mean age was 69.70 (SD = 7.48) for all community samples, 66.13 (SD = 4.37) for young-old adults, and 80.17 (SD = 4.11) for old-old adults. The education level, income level, and marital/living status of the whole community sample in terms of age and gender groups were summarized in Table 1.

### 3.2. SCD Mean Score Differences between Age and Gender Groups

To compare the mean SCD differences between the age and gender groups, Table 2 presents the mean (SD) SCD for 681 old adults stratified via each group with other demographic variables controlled through one-way ANOVA. The results showed that the females have higher mean SCD than males in old adults. Old-old adults have significant higher SCD scores than young-old adults. However, under the male cohort, there was no significant SCD difference between old-old males and young-old males.

### 3.3. SCD Score and Prevalence of Mental Fatigue Symptoms

**Figure 1:**
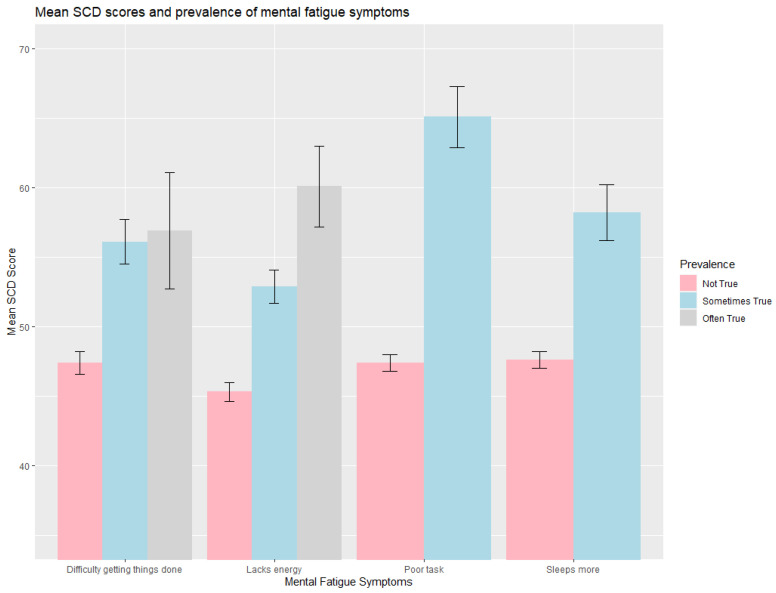
Means of SCD between old adults with and without mental fatigue symptoms. The “Sometimes True” and “Often True” of Poor task and Sleeps more were combined due to the infrequent of “Often True” (<5%). Error bar: 95% confidence interval.

The means of SCD grouped using each mental fatigue symptom were compared via one-way ANOVA. Poor task performance and Sleeps more were recoded into binary categories of Not True/Sometimes True (instead of Not True/Sometimes True/Often True) because of the infrequent occurrence of “Often True” (<5%). Figure 1 shows the means of SCD grouped using mental fatigue symptoms with 95% confidence intervals. Overall, the prevalence of mental fatigue symptoms increased the means of SCD among old adults. The mean SCD and ANOVA were detailed in Table A1 (Appendix A).

### 3.4. Factors Associated with SCD among Old Adults

To conclude, mental fatigue symptoms were significant predictors of SCD for old adults and were significant within each subgroup. Table 3 and Table 4 present the results of general linear regression models for old adults, broken down for the gender and age groups to examine the independent effect of each potential variable on SCD and adjusted for other variables. Females are associated with higher SCD scores than their male counterparts. The SCD score was 12 times higher on average for old adults who often had Poor task than those who had non-prevalence of the symptoms. Old adults who often had Sleeps more scored 3.79 higher on SCD on average than their counterparts. Positive associations were found between SCD and higher scores of Difficulty getting this done (β = 2.74, *p* < 0.001) and Lacks energy (β = 4.49, *p* < 0.001).

Within the gender cohort, the SCD among males could be predicted by the prevalence of Difficulty getting things done (β = 2.39, *p* < 0.001), Poor task (β = 11.44, *p* < 0.001), and Lacks energy (β = 4.26, *p* < 0.001). Among females, SCD was positively associated with higher scores in all mental fatigue items (*p* < 0.001) and negatively associated with marital/living status while controlled for all other variables.

The income and gender are associated with SCD among young-old adults. Young-old females and young-old adults with a low-income level tend to score lower on SCD. SCD was positively associated with higher scores of each mental fatigue problem at a significant level of *p* < 0.001. Among old-old adults aged 75 and above, SCD was associated with higher scores of Poor task (β = 11.3065, *p* < 0.001), Sleeps more (β = 3.872, *p* < 0.05), and Lacks energy (β = 3.490, *p* < 0.001). In contrast with the young-old cohort, Difficulty getting things done had no significant relationship with SCD among the old-old cohort.

## 4. Discussion

As the global population shifts to an older demographic, SCD has become an increasing issue [1,27]. Mental fatigue has been a common complaint of elderly individuals but has not been subject to much attention [24]. This study utilized cross-sectional survey data to investigate the progression of SCD with various factors and the relationship between mental fatigue and SCD in elderly Chinese populations. We report several key findings. First, SCD significantly increases with age from the young-old cohort to the old-old cohort. Second, females exhibit significantly higher SCD compared to their male counterparts for both age cohorts. Last and most importantly, mental fatigue factors are significantly and positively associated with SCD. These findings are crucial to developing early intervention strategies to preserve the mental health of an increasingly aging population.

The first objective of this study was to determine the progression of SCD in association with age and gender. Results indicate that SCD incidence increases with age, as shown by the significantly higher SCD scores in the old-old adults compared to the young-old adults. This is consistent with the current literature that supports the view that cognition declines with age [6,8,19,25]. This study is crucial to shed light on the SCD differences based on sex—our data supports the incidence of SCD to be higher in women. The current literature is mixed on the gender differences associated with SCD [14,29,36,37,38,39]. Schliep et al. found no significant difference in the prevalence of SCD between elderly men and women [36]. Wang et al. found that American elderly males exhibit worse cognition, while Levine et al. found that American elderly females exhibit faster cognitive decline [37,38]. However, there has been ample evidence in the literature supporting the idea that being female is associated with a higher level of SCD in the Chinese cohort [14,29,39]. The exact reason of the gender factor of SCD is not yet clear, and more research is needed to fully understand the underlying mechanisms. The loss of estrogen in women might be one of the possible explanations because estrogen facilitates higher cognitive functions by exerting effects on brain regions [40]. Reuben et al. found complaints of cognitive decline increased across the menopause transition, and women taking estrogen-decreasing treatments also had increased cognitive complaints and reduced working memory and executive function [41]. It is also important to consider psychological factors, such as depression, as another possible explanation of a higher prevalence of SCD in females. Females in China were observed to have higher prevalence of depression than males [42]. Furthermore, depression was found to be associated with SCD among Chinese old adults [43,44]. Lv et al. found that depression is associated with SCD solely among the Chinese female gender [44], suggesting that depression is another possible reason for the higher prevalence of SCD among females in China.

Our study found that high income is the protective factor of SCD in the young-old cohort. The current literature demonstrated similar results: cognitive decline is more likely to occur in young-old people living in lower socioeconomic areas compared to the high socioeconomic areas in southern Brazil and Latino [45,46]. Instead of stratifying the socioeconomic status by area, our finding demonstrates the association within the mixed community samples, which enhances the importance of income as a crucial factor associated with cognitive decline among the elderly in their 60s. However, the results should be treated with caution because of social policy and cultural effects in China. Financial resources may come from health insurance, pensions, and family support. It is important to note that the old adults are less likely to be covered by pensions and health insurance in rural areas, and it is common for old adults to receive financial support from family members, which does not fall under the definition of monthly income [39].

Stable marital relationships can ensure daily care and emotional sustenance for older adults, while those who are separated, divorces, windowed, or never married may suffer from loneliness, insecurity, feeling a lack or loss of relationships, making them more vulnerable to cognitive decline [39,47,48,49]. The current literature supports the importance of marital/living status—Nerobkova et al. suggests that the status of being unmarried, divorced, or widowed was associated with the risk of cognitive decline [50], and Liu et al. found evidence that, compared to married counterparts, divorced and widowed elders had higher odds of dementia and cognitive impairment in the United States, but that the results did not vary by gender [51]. Meanwhile, Feng et al. found that the association between marital status and cognitive decline was statistically significant only for men among community-dwelling Chinese older adults [52]. However, our study found that marital/living status is associated with SCD solely among Chinese females. Therefore, the findings should be interpreted with caution and more comprehensive studies regarding marital status and cognitive decline with gender differences are needed in the future.

Mental fatigue is a common complaint among elderly individuals and manifests as heightened feelings of tiredness, increased reaction times, and reduced physical energy [23,24]. To our knowledge, no study has focused on the association between mental fatigue and SCD in elderly Chinese populations. Our research shows a positive association between mental fatigue symptoms and the incidence of SCD in elderly adults. Among males, SCD was positively associated with higher scores in several mental fatigue symptoms such as: Difficulty getting things done, Poor task, and Lacks energy. Among females, SCD was positively associated with all mental fatigue symptoms. This aligns well with the literature linking each of these symptoms to mental fatigue. It is well known that physical activity can improve cognitive function [53]. However, mental fatigue impairs physical performance and increases physical fatigue, which contributes to the difficulty of completing tasks, a lower task completion quality, and a general lack of energy [27,54]. In addition, Suh et al. showed that longer sleep duration in non-demented elderly samples experienced a greater risk of cognitive decline [55]. This research further corroborates the symptomatic expression of mental fatigue in elderly individuals. Previous research has shown the association between various mental stressors and cognition decline in various adult samples, but this research clarifies and our current study further clarifies and advances our understanding of mental fatigue being a target stressor for SCD in elderly [25,31,32].

The findings should be interpreted considering the limitations of the study. First, this cross-sectional study measures the prevalence of the cognitive decline problem based on the survey that collected both explanatory variables and outcomes at the same point in time without a prospective or retrospective follow-up. Hence, demonstrating a causal relationship is not possible at this time. Second, the community sample consisted of older adults from Shanghai, which is one of the most well-developed cities in China. This limits the generalization of the findings to other geographic regions, particularly rural and ethnically diverse areas. Third, the OASR, which was developed based on Western samples, possibly does not capture the full spectrum of behaviors and traits specific to Chinese culture and the Chinese population.

Despite these limitations, our study shows that SCD worsens with age, is significantly associated with the female gender, and is positively associated with mental fatigue symptoms in Chinese elderly populations. While this study showed significantly higher SCD incidence in women, the current literature is mixed on the differences in SCD prevalence between gender, indicating a need for further research [36,37,38]. Future directions include continued follow-ups with the cohort and testing possible intervention strategies to slow the progression of SCD and reverse the symptoms of mental fatigue in these elderly samples. Such data will also help clarify possible additional risk factors for SCD and the most effective approaches for improving mental fatigue and slowing SCD. Our current and future findings may have important implications for the early detection of SCD and prevention of more serious cognitive impairments, such as Alzheimer’s disease, in increasingly aging populations.

## 5. Conclusions

To our knowledge, the present study is the first to explore the association between mental fatigue and SCD among the elderly Chinese population. Results showed that SCD increased with age and had a higher prevalence in the female gender compared to the male gender. SCD was positively associated with higher scores in these mental fatigue symptoms: Difficulty getting things done, Poor task, Sleeps more, and Lacks energy. This study contributes evidence for mental fatigue to be targeted in Alzheimer’s disease and cognitive impairment prevention and for improving the cognition and mental health of elderly individuals. Further examination is needed to develop effective treatment methods for various aspects of mental fatigue and SCD in order to provide long-term mental protection for an increasing number of elderly individuals.

## Figures and Tables

**Table 1:** Sample characteristics by age and gender.

Variables	Young-Old (N = 508)	Old-Old (N = 173)	Males (N = 306)	Females (N = 375)	Total (N = 681)
Age	66.13 (4.37)	80.17 (4.11)	70.69 (7.65)	68.89 (7.25)	69.7 (7.48)
Young-Old	--	--	212 (69.28%)	296 (78.93%)	508 (74.60%)
Old-old	--	--	84 (27.45%)	79 (21.07%)	173 (25.40%)
Gender					
Males	212 (41.73%)	94 (54.34%)	--	--	306 (44.93%)
Females	296 (58.27%)	79 (45.66%)	--	--	375 (55.07%)
Education Level					
High-Edu	182 (35.83%)	74 (42.77%)	127 (41.50%)	129 (34.40%)	256 (37.59%)
Low-Edu	326 (64.17%)	99 (57.23%)	179 (58.50%)	246 (65.60%)	425 (62.41%)
Income Level					
High-income	251 (49.41%)	95 (54.91%)	179 (58.50%)	167 (44.53%)	356 (52.28%)
Low-income	257 (50.59%)	78 (45.09%)	127 (41.50%)	208 (55.47%)	335 (49.19%)
Marital/Living Status					
Live w. Spouse/Partner	393 (77.36%)	93 (53.76%)	244 (79.74%)	242 (64.53%)	486 (71.37%)
Other	115 (22.64%)	80 (46.24%)	62 (20.26%)	133 (35.47%)	196 (28.78%)

**Table 2:** The cross-table of mean SCD comparison between age and gender groups.

		All		Young-Old		Old-Old	
Group	*n*	Mean (SD)	*n*	Mean (SD)	*n*	Mean (SD)	F(p) ^2^
All	681	50.00 (10.05)	508	49.49 (9.96)	173	51.48 (10.21)	7.044 (0.008) **
Males	306	48.69 (8.83)	212	48.25 (8.25)	94	49.68 (9.98)	1.728 (0.190)
Females	375	51.06 (10.85)	296	50.38 (10.95)	79	53.62 (10.13)	5.802 (0.016) *
F (p) ^1^		9.759 (0.002) **		5.881 (0.015) *		6.583 (0.011) *	

^1^ Means were compared via one-way ANOVA, with covariance age, income, education, and marital/living status; ^2^ means were compared via one-way ANOVA, with covariance gender, income, education, and marital/living status; * = *p* < 0.05, ** = *p* < 0.01.

**Table 3:** Adjusted general linear regression for factors associated with SCD among old adults and in each gender cohort.

	Total			Males			Females		
Variables	β (SE)	95% CI	*p*	β (SE)	95% CI	*p*	β (SE)	95% CI	*p*
Gender	1.63 (0.533)	(0.583, 2.677)	0.002 **	--	--	--	--	--	--
Age	0.07 (0.036)	(−0.005, 0.135)	0.071	0.06 (0.048)	(−0.034, 0.153)	0.209	0.05 (0.053)	(−0.051, 0.157)	0.317
Education	−0.15 (0.175)	(−0.488, 0.197)	0.404	−0.31 (0.238)	(−0.782, 0.156)	0.190	−0.02 (0.250)	(−0.510, 0.474)	0.943
Income	−0.52 (0.543)	(−1.588, 0.544)	0.337	−0.98 (0.753)	(−2.465, 0.499)	0.193	−0.26 (0.764)	(−1.762, 1.241)	0.733
Marital/living status	−0.78 (0.600)	(−1.955, 0.400)	0.195	1.11 (0.912)	(−0.684, 2.907)	0.224	−1.61 (0.804)	(−3.188, −0.027)	0.046 *
Difficulty getting things done	2.74 (0.449)	(1.861, 3.623)	0.000 ***	2.39 (0.628)	(1.150, 3.620)	0.000 ***	2.98 (0.626)	(1.753, 4.214)	0.000 ***
Poor task	12.00 (0.817)	(10.392, 13.601)	0.000 ***	11.44 (1.105)	(9.268, 13.618)	0.000 ***	12.12 (1.217)	(9.731, 14.517)	0.000 ***
Sleeps more	3.79 (0.678)	(2.459, 5.121)	0.000 ***	1.45 (0.903)	(−0.330, 3.224)	0.110	5.51 (1.026)	(3.496, 7.530)	0.000 ***
Lacks energy	4.49 (0.406)	(3.689, 5.283)	0.000 ***	4.26 (0.580)	(3.117, 5.398)	0.000 ***	4.512 (0.559)	(3.413, 5.610)	0.000 ***

β = the change in SCD score when the variable increase by one unit, controlled for all other variables. SE = Standard error. 95% CI = 95% confidence interval; * = *p* < 0.05, ** = *p* < 0.01, *** = *p* < 0.001.

**Table 4:** Adjusted general linear regression for factors associated with SCD in age cohorts.

	Young-Old Adult			Old-Old Adult		
Variables	β (SE)	95% CI	*p*	β (SE)	95% CI	*p*
Gender	1.46 (0.557)	(0.369, 2.556)	0.009 **	2.52 (1.478)	(−0.406, 5.438)	0.091
Age	0.09 (0.059)	(−0.024, 0.208)	0.120	0.16 (0.177)	(−0.193, 0.509)	0.375
Education	−0.15 (0.182)	(−0.505, 0.211)	0.421	−0.38 (0.480)	(−1.329, 0.567)	0.428
Income	−1.17 (0.564)	(−2.275, −0.058)	0.039 *	1.95 (1.483)	(−0.985, 4.877)	0.192
Living status	−0.86 (0.663)	(−2.164, 0.440)	0.194	−0.35 (1.470)	(−3.258, 2.551)	0.810
Difficulty getting things done	3.05 (0.484)	(2.100, 4.000)	0.000 ***	1.68 (1.133)	(−0.563, 3.916)	0.141
Poor task	12.26 (0.916)	(10.457, 14.055)	0.000 ***	11.31 (1.889)	(7.573, 15.040)	0.000 ***
Sleeps more	3.61 (0.736)	(2.169, 5.059)	0.000 ***	3.87 (1.656)	(0.599, 7.145)	0.021 *
Lacks energy	4.90 (0.441)	(4.036, 5.767)	0.000 ***	3.49 (1.001)	(1.512, 5.467)	0.000 ***

β = the change in SCD score when the variable increase by one unit, controlled for all other variables. SE = Standard error. 95% CI = 95% confidence interval; * = *p* < 0.05, ** = *p* < 0.01, *** = *p* < 0.001.

## Data Availability

Data available upon request.

## Appendix A

**Table A1:** Means of SCD Grouped by Age, Gender, and Mental Fatigue Symptoms.

Variables		N	Mean (SD)	*p*
Difficulty getting things done	Not True	480	47.4 (8.18)	<0.001 ***
	Sometimes True	156	56.1 (10.4)	
	Often True	45	56.9 (13.8)	
Poor task	Not True	581	47.4 (7.19)	<0.001 ***
	Sometimes True	100	65.1 (11.1)	
Sleeps more	Not True	529	47.6 (7.52)	<0.001 ***
	Sometimes True	152	58.2 (13.00)	
Lacks energy	Not True	332	45.3 (6.52)	<0.001 ***
	Sometimes True	274	52.9 (9.76)	
	Often True	75	60.1 (12.50)	

The means of SCD were compared using one-way ANOVA. *** = *p* < 0.001.
